# Medical Plaster Enhancement by Coating with *Cistus* L. Extracts within a Chitosan Matrix: From Natural Complexity to Health Care Simplicity

**DOI:** 10.3390/ma14030582

**Published:** 2021-01-27

**Authors:** Monika Haponska, Paulina Modrakowska, Karolina Balik, Anna Bajek, Anne Coloigner, Bartosz Tylkowski, Marta Giamberini

**Affiliations:** 1 Eurecat, Centre Tecnològic de Catalunya, Marcel·lí Domingo s/n, 26-43007 Tarra-gona, Spain; monika.haponska@eurecat.org; 2 Department of Tissue Engineering, The Ludwik Rydygier Collegium Medicum, Nicolaus Copernicus University, 87-100 Torun, Poland; paulina.modrakowska@gmail.com (P.M.); karolinabalik@gmail.com (K.B.); a_bajek@wp.pl (A.B.); 3 Department of Chemical Engineering, Universitat Rovira i Virgili, Av. Països Catalans, 26-43007 Tarra-gona, Spain; anne.coloigner@estudiants.urv.cat

**Keywords:** medical plaster, carboxymethyl chitosan, biologic active compounds, radiation dermatitis

## Abstract

Our investigation was focused on the preparation and characterization of novel plasters based on Carboxymethyl Chitosan derivative (CMC), to be used for the treatment of radiation dermatitis with Biologic Active Compounds (BACs) in a moist wound-healing environment. After performing the extraction and characterization of BACs from *Cistus* L., we optimized the BACs/CMC solution for subsequent plaster preparation. Then, plasters were prepared by dip-coating with a different number of layers, and we characterized them by Environmental Scanning Electron Microscopy (ESEM), Contact Angle (CA) and release tests in water for 24 h. Taking into account the flexibility of the plasters and the amount of released BACs after 24 h, the sample obtained after two dip-coating steps (2La) appeared promising in regard to comfortable mechanical properties and active principles administration. The 3-(4,5-dimethylthiazol-2-yl)-2,5-diphenyltetrazolium bromide (MTT) test performed on keratinocytes cultured in standard medium shows that cells treated with released extract from 2La start to proliferate, extend cellular viability and form colonies typical for epidermal cells.

## 1. Introduction

Polyphenols are a well-known class of substances that possess at least one aromatic ring containing at least one hydroxyl group and/or other active residues as methyl or acetyl [1,2,3]. They are widely contained in plants and can be classified into the following groups: phenolic acids, flavonoids, lignans, curcuminoids, stilbenes, and tannins [4]. Their pattern and substitution degrees determine a broad spectrum of biological properties such as free radical scavenging, metal-chelating, antimicrobial, immunostimulating, wound-healing and chemopreventive activities [5,6,7,8,9]. For instance, their natural antioxidant activity is able to inhibit oxidative chain reactions in human skin and can therefore prevent some skin diseases, premature ageing and skin damage, including wounds and burns [10]. In addition, they have been reported to inhibit the skin enzymes responsible for catalyzing the hydrolysis of collagen, elastin fibers and hyaluronic acid in the dermis [7,11]. It has been found that plant phenolics can be beneficial either by the diet or through skin application. Actually, the delivery of polyphenols through the skin is currently receiving considerable attention with the aim of protecting skin from oxidative stress, premature ageing or other diseases.

Nevertheless, since skin works as a barrier, most of the active ingredients applied topically on the skin exhibit a low natural permeability. For this reason, many physical and chemical methods such as, for instance, occlusive dressings or reversible chemical modification of the skin barrier, have been developed to enhance the permeability of the active compounds [1,2]. In addition, topical absorption as well as performance of polyphenol-containing formulations can be improved by means of encapsulation through liposomes, nanoparticles, nanocrystals, etc. [3]. As an alternative, phenolic compounds can also be delivered directly to the irritated skin by means of textiles [4].

Radiation dermatitis is a common side effect of radiotherapy, which is one of the most frequently used treatments for cancer, together with chemotherapy and surgery. This skin reaction can range from a mild erythema to moist desquamation and even ulceration. It has been reported that approximately 85–87% of patients treated by radiotherapy experience a moderate to severe skin reaction [5,6] and 10–15% of them will progress to moist desquamation [7]. Skin dermatitis generally arises within a few days or weeks after the beginning of radiotherapy, depending on the dose of radiation and individual skin sensitivity. Furthermore, some chemotherapy agents are radiosensitizers (e.g., 5-Fluorouracil, Mitomycin C, Cisplatin) and can therefore lead to a more severe skin reaction. The skin can peel in a dry way, similar to an old sunburn, or in a wet way, like a blister. When the blister opens, the exposed raw area can run into a severe infection if it is not properly treated. Therefore, it is desirable to promote a moist wound-healing environment, in the stages where skin is broken.

*Cistus Ladanifer* (*Cistus* L.) species have been widely applied in folk medicine. They contain gallic acid, rutin, different flavonoid aglycones, and flavan-3-ols, as well as catechin, epicatechin, gallocatechin, gallocatechin-3-gallate and oligomeric procyanidin B1 and B3 as Biologically Active Compounds (BACs) [8]. In a previous paper, we showed that BAC content and activity are preserved when a steam sterilization process is employed, thus expanding the application opportunities in Medicine for these natural compounds [9].

Chitosan is a polysaccharide which is very attractive for food, medical and pharmaceutical applications, given its outstanding properties such as non-toxicity, superb biodegradability, high biocompatibility, abundant availability and low cost. Carboxymethyl Chitosan derivative (CMC) exhibits many advantages to be used for producing membranes, which contain BACs such as polyphenols. On the one hand, this chitosan derivative has improved water solubility [10]; on the other hand, it has been also demonstrated that the antibacterial activity is higher for CMC than underived chitosan [11]. Studies on CMC also revealed that its amino and hydroxyl groups, as well as its lower number of hydrogen interactions, lead to better antioxidant activity, which is of importance in the treatment of various pathological phenomena that are related to the presence of reactive oxygen species. Chitosan derivatives, and particularly the CMC, constitute promising polymers in different biomedical fields, thanks to their antimicrobial, anti-cancer, anti-tumor, anti-oxidant and antifungal properties [12]. 

In this paper, we explored the possibility to deposit CMC/BACs onto a cotton plaster by dip-coating; the resulting plaster could be applied for care of broken skin when radiation dermatitis arises. We extracted BACs from *Cistus* L. and studied the optimal CMC/BACs proportion for subsequent dip-coating steps; afterwards, we examined the characteristics of the resulting plasters. The obtained plasters should be flexible, easy to handle and able to release the active principles in an aqueous environment in reasonable time.

## 2. Materials and Methods

### 2.1. Materials

Folin–Ciocalteu’s reagent, sodium carbonate, aluminium chloride, gallic acid, and quercetin were purchased from Sigm-Aldrich (Sigma Aldrich Química, Madrid, Spain). All reagents were used without further purification. The *Cistus* L. was cultivated in Turkey and distributed by Radix-Bis Sp. z o.o. (Rotmanka, Poland). Carboxymethyl chitosan (CMC) was provided by Santa Cruz Biotechnology (Dallas, TX, USA). 

The powdered plant material from *Cistus* L. was extracted with liquid–solid ratio 15:1 (g/g), earlier determined as optimal to the experiments [13]. After 24 h of extraction by mixing, the mixture was filtered by gravity filtration. Extract was stored in the dark at 4 °C, in order to protect it from photodegradation [14].

Then, 5 wt.% aqueous solution of carboxymethyl chitosan was obtained by gradual addition of a weighed amount of polymer to deionized water under magnetic stirring. After 3 h of mixing, the solution was stored in the dark at 4 °C.

Membranes were prepared by casting the required aqueous solution on a Teflon support and subsequent solvent evaporation 24 h at 50 °C; we used 5 wt.% CMC solutions diluted 4:1, 3:2 and 3:1 with water or with the *Cistus* L. extract, respectively.

Solution A for dip-coating was made by mixing the previously prepared 5 wt.% chitosan derivative and *Cistus* L. extract in ratio 4:1 under magnetic stirring. The solution was mixed for 3 h and then left standing for 1 h to prevent the appearance of bubbles.

**Figure 1:**
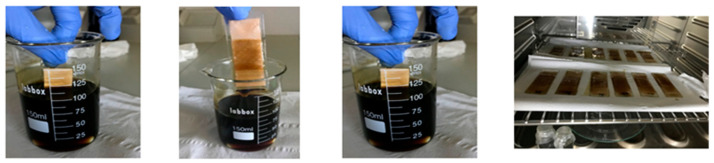
Plaster enhancement. From left to right: dipping in solution A for 30 s, taking out and turning 180°, dipping for additional 30 s and drying for 24 h at 40 °C.

Water-resistant plasters (Hansaplast Universal, Barcelona, Spain) were cut into 2 cm × 2.5 cm pieces and subsequently stuck to a glass cover with tape. They were subsequently dipped in solution A for 30 s, taken out, turned 180° and dipped for an additional 30 s (Figure 1). This procedure was repeated from one to three times on each plaster, finally yielding plasters named 1La, 2La, 3La, 4La, with number of coated layers equal to 1, 2, 3 or 4, respectively. Twelve plasters were prepared, three samples for each number of layers. Next, they were dried at 40 °C for 24 h in a normal oven. Each sample was weighed before dip-coating and after dip-coating followed by drying.

### 2.2. Analytical Techniques

The total phenolic content (TPC) was determined spectrophotometrically [15]. *Cistus* L. extract was diluted 10-fold, and 0.5 mL of the diluted sample was added to a flask, containing 10 mL of H_2_O. Afterwards, 0.5 mL of Folin–Ciocalteu’s reagent (FC) was added. After 5 min, 8 mL of 7.5% aqueous Na_2_CO_3_ solution was added to the mixture and subsequently stored for two hours in the dark. The concentration of released phenols was determined by UV-Vis spectroscopy at 765 nm by means of UV-1800 Shimadzu apparatus (ThermoFisher Scientific, Waltham, MA, USA). The results were calculated as Gallic Acid Equivalents (GAE), using a standard curve obtained from previous experiments: Abs = 0.1214x + 0.1142, where Abs is the absorbance of the sample at 765 nm and x is the phenolic concentration in µgGAE/mL (R^2^ = 0.9988). All samples were analyzed in triplicate. The blank was obtained with the same procedures, by using 0.5 mL of H_2_O instead of the *Cistus* L. diluted sample.

Total flavonoid content (TFC) was determined spectrophotometrically [16]. 0.5 mL of the sample was mixed with 0.5 mL of 2% (*w*/*v*) ethanolic solution of AlCl_3_. The mixture was stored for one hour in the dark. The concentration of released flavonoids was determined by UV-Vis spectroscopy at 420 nm using a UV-1800 Shimadzu apparatus (ThermoFisher Scientific, Waltham, MA, USA). The results were calculated as Quercetin Equivalents (QE), using a standard curve obtained from previous experiments: Abs = 0.0674x + 0.0182, where Abs is the sample absorbance at 420 nm and x is the phenolic concentration in µgQE/mL (R^2^ = 0.9955). All samples were analyzed in triplicate.

The radical scavenging activity of the extract was determined by DPPH photometric assay at 515 nm [17]. Methanolic solutions of 2,2-difenil-1-picrylhydrazyl (DPPH) were used as standard to build the calibration curve (Supplementary material, Figure S1): Abs = 0.0145x + 0.0716 (R^2^ = 0.9823), where Abs is the sample absorbance at 515 nm and x is DPPH concentration. A total of 0.1 mL of the extract sample was added to 3.9 mL of 60 µM solution of DPPH in methanol. DPPH concentration was determined at time 0 and after 20 min reaction in the dark at room temperature to form the stable molecule DPPH-H, by monitoring the absorbance at 515 nm. Methanol was used as blank solution. The radical scavenging activity (RSA) was calculated according to the equation:

%RSA = ([DPPH]_0_ − [DPPH]_20_)/[DPPH]_0_ × 100 (1)

where [DPPH]_0_ and [DPPH]_20_ are the concentrations of DPPH at time 0 and after 20 min of reaction, respectively.

In order to determine the release of the active compounds to the water, the cotton layer of the plasters was separated from the tape and was immersed in a small glass jar. A total of 6 mL of water was added to the jar to completely cover the cotton sample. After 30 min, 1 h, 10 h, 20 h and 24 h of incubation, respectively, 1 mL of solution from the jar was withdrawn in order to measure TFC and TPC as previously described. Next, the cotton samples were removed from the jar and dried at 40 °C for 24 h. The sample weight was measured in each step of the experiment. 

### 2.3. Characterization Techniques

Wettability of plasters with different number of layers of *Cistus L.* extract-chitosan derivative solution was determined by static contact angle measurements by means of Dataphysics PCA 15EC DataPhysics Instruments GmbH, Filderstadt, Germany). A 3 μL droplet of Milli-q water was placed on the surface of the membrane and the contact angle was calculated from a digital image by SCA software included in the apparatus. Contact angle values were taken as the average of three measurements. An untreated plaster was used as a blank.

Dip-coated plaster morphology was characterized by environmental scanning electronic microscopy (ESEM), using an FEI Quanta 600 (ThermoFisher Scientific, Waltham, MA, USA). Samples were prepared by cutting the plasters with scissors and sticking cut plasters with conductive adhesive carbon tabs to the sample holders. Plaster thickness was measured by ESEM on the sample cross-section at least in three points.

X-ray diffraction (XRD) analyses measurements were taken using a Bruker-AXS D8-Discover diffractometer (Bruker, Wien, Austria), equipped with parallel incident beam (Göbel mirror), vertical θ-θ goniometer, XYZ motorized stage and with a GADDS (General Area Diffraction System). An X-ray collimator system close to the sample allows one to analyze areas of 500 μm. The X-ray diffractometer was operated at 40 kV and 40 mA to generate CuKα radiation. 

3-(4,5-dimethylthiazol-2-yl)-2,5-diphenyltetrazolium bromide (MTT) assay: keratinocytes (Ker-CT) cells (ATCC, Manassas, VA, USA) were seeded in a 96-well plate (BD Falcon, Tewksbury, MA, USA) at a density of 5 × 105 cells/well, cultured in KBM Gold (Keratinocyte Basal Growth Medium) (Lonza, Switzerland) for 24 h and treated with total polyphenols and flavonoids released from 2La plasters after 10 and 24 h as well as from Cistus L. herb. Additionally, all extracts were added to the wells without Ker-CT cells to serve as a blank. After 24 h, cells were washed with HBSS (Hank’s Balanced Salt Solution) (ATCC, USA) and 50 µL of MTT solution (Sigma-Aldrich, Madrid, Spain) was added (1 mg/mL in MEM without phenol red). After 3 h of incubation, formazan crystals were dissolved in 100 µL DMSO. The absorbance was read at 570 nm (Multiskan Sky Microplate Spectrophotometer) (ThermoFisher Scientific, Waltham, MA, USA). The well containing DMSO was treated as a blank. The images were taken before the MTT assay with the use of an inverted phase contrast microscope (CKX53FL) (Olympus, Tokyo, Japan) with a dedicated camera (UC90) (Olympus, Tokyo, Japan) and with a 10x magnification lens (UPLFLN10X2PH) (Olympus, Tokyo, Japan). The images were analyzed with Cellsens dimensions (Olympus, Japan). One-way ANOVA—Sidak’s test—was used. * *p* ≤ 0.05; ** *p* ≤ 0.01; *** *p* ≤ 0.001. The statistical analysis was performed using GraphPad Prism 7.0—trial version (GraphPad Software, San Diego, CA, USA). The studies with the cells were carried out with permission and according to the Local Bioethical Committee from Collegium Medicum of Nicolaus Copernicus University in Torun, Poland. The Ker-CT (ATCC^®^ CRL-4048™) cell line was obtained from the ATCC Company based in Manassas, VA, USA.

## 3. Results and Discussion

### 3.1. BAC Content and Radical Scavenging Activity (RSA)

The total phenolic and flavonoid contents were determined as described in the experimental part and gave the following results: as for polyphenols, gallic acid equivalents = 142 μg/mL; as for flavonoids, quercetin equivalents = 483 μg/mL. Though higher yields could be obtained by using ethanolic solutions [18], extraction was performed in water, in order to prepare an extract able to completely solubilize carboxymethyl chitosan [19]. It has been recently reported that extraction of *Cistus* L. in water can lead to an amount of gallic acid equivalents comprised between 54 and 219 μg/mL, depending on the used species and the season of collection [20]; therefore, we considered our result acceptable.

We also determined the radical scavenging activity (RSA) of the extract by means of DPPH assay. The relative parameter RSA only shows the ability of the sample, at a fixed concentration, to reduce or not reduce the radicals. In many cases, the increase in the concentration of the antioxidant leads to the increase in these relative indices [21]. When the radical DPPH is scavenged by antioxidants from donation of hydrogen to form the stable molecule DPPH-H, the color of the solution changes from purple to yellow and the absorbance at 515 nm gradually decreases. In our case, after 20 min of reaction with the extract, DPPH concentration changed from 4.83 to 2.46 μg/mL, giving RSA = 49%. This rapid decrease in DPPH concentration indicates that there is a powerful antiradical activity.

### 3.2. Plasters Preparation

The following step of our research concerned the optimization of the extract/polymer solution to be used for plaster coating. This aspect was essential to obtain flexible membranes in combination with the cotton plaster. For this purpose, we prepared several CMC/BACs solutions and to use them for membrane casting onto a teflon support. We took into consideration two aspects:

- Maximum amount of CMC that could be used;
- Effect of CMC/BACs proportion on the final mechanical properties of the cast membranes.

As far as the first point is concerned, we found that the upper limit of CMC concentration to be used is 5 wt.%. In the case of CMC higher amounts, the resulting solution appeared too viscous and unsuitable for the following dip-coating procedures. Therefore, 5 wt.% of CMC was the concentration not to be exceeded to prepare polymer solutions. Actually, it was reported that the apparent viscosity of CMC solutions increases with increasing concentrations. This rise in the apparent viscosity is due to the increase in the intermolecular interactions between CMC molecules. Moreover, it was reported that above a critical concentration equal to approx. 1 wt.%, CMC, the solution microstructure changes from a semi-diluted non-entangled solution to a semi-diluted entangled network. This change is reported as being responsible for a thixotropic behavior [22].

Chitosan derivatives can establish many interactions with the solvent and the *Cistus L*. extract itself containing BACs, which can eventually modify the quality of the cast membranes. To determine the optimal CMC/BACs proportion to be used for further experiments, we mixed 5 wt.% CMC solution with the extract in different proportions, i.e., 4:1, 3:1, 3:2; in a following step, solutions were made under the same conditions, but replacing the extract with water. Finally, membranes were cast out of all the prepared solutions. In this way, the influence of the extract on the mechanical properties of the resulting membranes could be evaluated.

We found that the membranes obtained out of the CMC/extract 3:1 and 3:2 solutions, respectively, were sticky, rigid and brittle, while the corresponding ones prepared with water instead of the extract could be peeled off the support and were flexible. 

We performed XRD analysis in order to establish whether the presence of BACs could induce crystallinity in the final membrane, thus increasing brittleness. In fact, the interactions that the polyphenols can establish are numerous, such as hydrogen bonding, hydrophobic, electrostatic and covalent interactions, etc. Chitosan can conjugate with polyphenols via strong electrostatic interactions with polyphenols [12]. Therefore, we compared the XRD of the membrane obtained out of the CMC/extract 3:2 with the one obtained by replacing the extract with water; moreover, we also examined the XRD of a film cast from the extract alone. 

**Figure 2:**
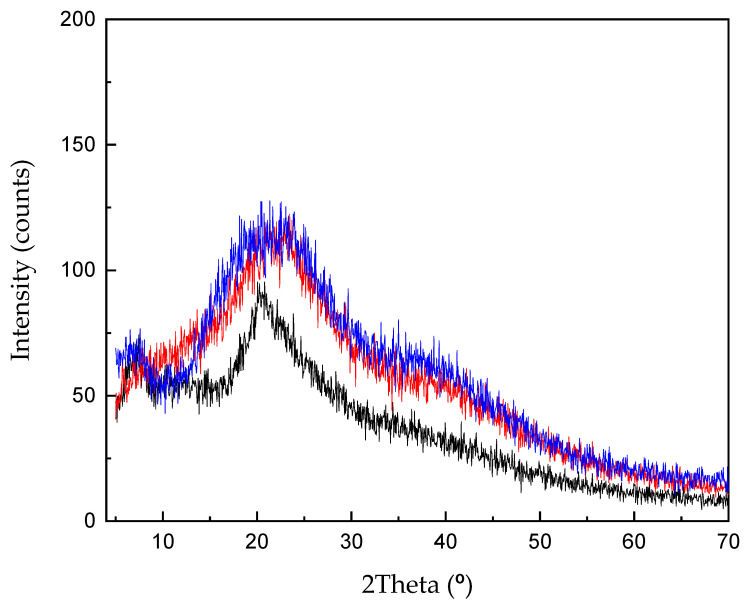
XRD diffraction pattern of the membranes obtained out of: Carboxymethyl Chitosan derivative (CMC)/extract 3:2 (red line); CMC/water 3:2 (black line); Extract alone (blue line).

Only broad halos, characteristic of amorphous structures, were shown by the three samples (Figure 2); the membrane obtained out of the CMC/extract 3:1 exhibited the same XRD pattern as CMC/extract 3:2. Therefore, the presence of crystallinity in the final membranes could be ruled out.

In the case of CMC/extract 4:1, the cast membrane could be easily removed from the support and resulted quite flexible. Therefore, the polymeric solution/BACs 4:1 appeared to be the most suitable for carrying out the next studies. Solution A was prepared, which contained 5 wt.% chitosan derivative and *Cistus L.* extract in ratio 4:1 (wt/wt), and used for the dip-coating of plasters. 

**Figure 3:**
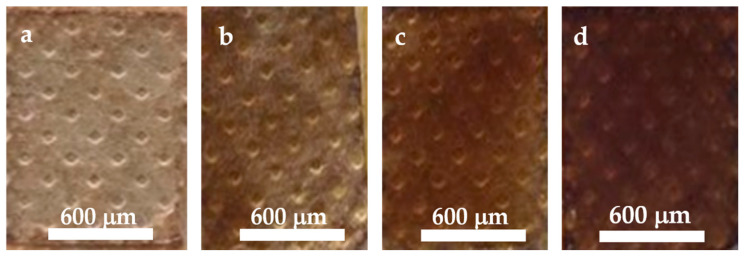
Plasters dip-coated in Solution A after drying: 1La, one layer (**a**); 2La, two layers (**b**); 3La, three layers (**c**); 4La, four layers (**d**).

The plasters were dip-coated one, two, three or four times for 30 s and subsequently dried at 40 °C for 24 h (samples 1La, 2La, 3La and 4La, respectively). The images of plasters after this procedure are shown in Figure 3.

### 3.3. Plasters Characterization

As for direct observation of the obtained plasters, the following remarks could be made: sample 1La exhibited unevenly distributed carboxymethyl chitosan/active compounds layer; on the other hand, the adhesive tape was undamaged and could be easily detached from the glass. In the case of sample 2La, a more uniform carboxymethyl chitosan/active compounds distribution on the plaster surface was put into evidence with a naked eye. However, the adhesive tape tends to crumble and some difficulties were encountered when detaching it from the glass. Samples 3La and 4La exhibited worse mechanical properties, since they easily crumbled and it was impossible to detach them from the glass support. All subsequent tests on samples 3La and 4La had to be performed with the aid of the glass support. 

**Figure 4:**
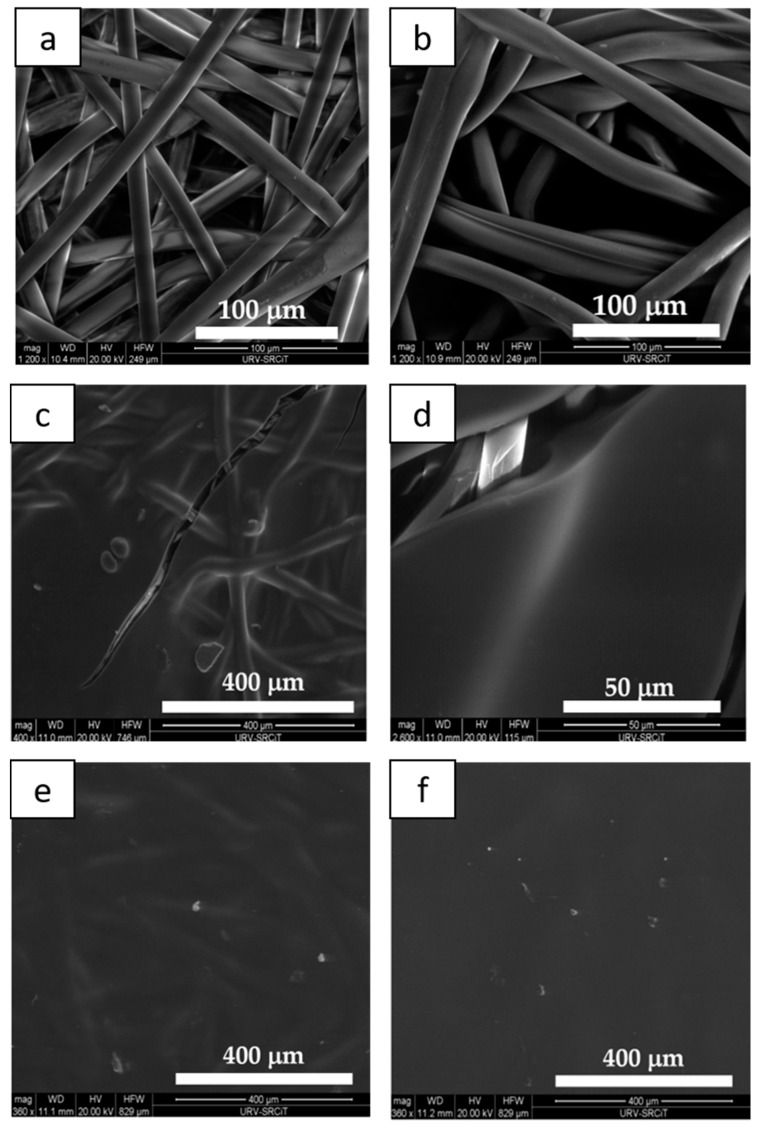
Environmental scanning electronic microscopy (ESEM) image of: a neat plaster (**a**); 1La sample (**b**); 2La sample (**c**) and its detail (**d**); 3La sample (**e**); 4La sample (**f**).

The distribution of carboxymethyl chitosan/active compounds on the plaster fibers was studied by ESEM. Figure 4 depicts the ESEM image of a neat plaster and 1La-4Lasamples (b).

The comparison of the two images puts into evidence that, in the case of one layer (sample 1La, Figure 4b), the chitosan solution containing BAC extract was unregularly distributed on the plaster fibers. Fiber thickness of the dip-coated plaster was measured and gave an average value 15.4 ± 1.2 μm over 20 fibers, which corresponds to a 1.6 μm increase with respect to the neat fibers. Otherwise, in the case of two layers (sample 2La), ESEM analysis showed that the dip-coating formed a film on the plaster surface (Figure 4c).

Plasters dip-coated three and four times, respectively, exhibited a thicker and more uniform film which hid the plaster fibers (Figure 4e,f); in the case of 4La sample, fibers could not be glimpsed anymore. The presence of a thicker CMC/BACs film is clearly responsible for the increased brittleness of the final plasters.

Plaster wettability was evaluated by Static Contact Angle (CA) measurements. For the sake of comparison, we also evaluated the wettability of a plaster obtained by dip-coating twice in 4:1 CMC/water, i.e., made under the same conditions, but replacing the extract with water (sample 2LaW). The results are shown in Table 1.

As expected, the coating with CMC solution determines a remarkable increase in the hydrophilicity of the plaster surface, which water CA passed from around 113°, clearly indicative of hydrophobicity, to 61° for the plaster coated by two layers of CMC solution. A further remarkable decrease in CA was found when the coating solution also contained BACs, i.e., CA reached a value as low as approximately 18° in 2La–4La samples. The CA of 1La resulted 9° ± 2, but it is not reported for direct comparison in Table 1, due to the fibrous surface of this sample (Figure 4b). Actually, quantitative wettability determination of fiber or yarns require much more careful analysis, since it is affected by factors such as complex spreading, capillary forces, pore size distribution, etc. [23,24]. On the other hand, the uniformly coated plasters 2La–4La exhibited good water wettability.

### 3.4. Release Experiments

**Figure 5:**
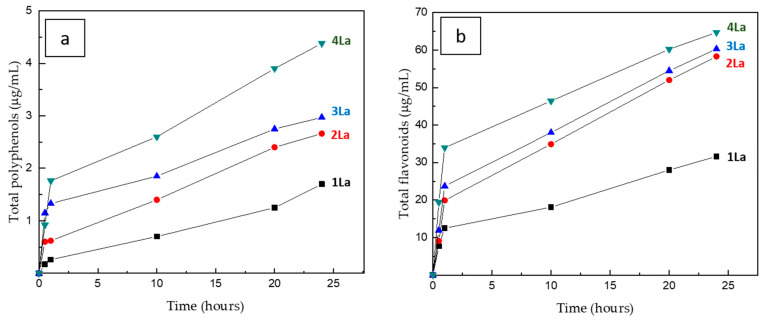
Kinetics of total polyphenols (**a**) and total flavonoids (**b**) released from plasters: 1La, 2La, 3La, and 4La.

BAC release was investigated in water as described in the Experimental Section. The results are depicted in Figure 5a,b, concerning total phenols and flavonoids release, respectively.

Polyphenols are generally considered moderately water-soluble compounds, while most flavonoids are present in plants bound to sugars in the form of β-glycosides, which are readily soluble in water. This fact clearly determines a big difference between their release kinetics, the flavonoids being much faster in passing into an aqueous environment. Nevertheless, neither for polyphenols nor flavonoids did the release kinetics reach a saturation after 24 h, as can be noticed from Figure 5a,b. The released amount is higher on increasing the number of layers coated on the plaster, as expected. In all cases, a “burst” release was observed in the first hour and can be attributed to the compounds present on the layer surface. We could not normalize the released amounts to the total polyphenols/flavonoids present on each plaster, because we could not determine it after the dip-coating process for the different coated plasters. However, we can give an extremely rough estimation for the worst case, i.e., by considering the determined total content in the dip-coating solution as the maximum amount of polyphenols and flavonoids potentially available for release from the plasters. This is clearly a huge underestimation of the release, since it neglects the fact that (a) only a limited portion of BAC solution is deposited onto the plaster surface by dip-coating and (b) the deposited amount depends on the number of layers and it is higher when more dip-coating steps are performed. Under this assumption, the released amount of polyphenols after 24 h results 6% for 1La, 9% for 2La, 10.5% for 3La and 15% for 4La samples, respectively; as for flavonoids, after 24 h 33% was released from 1La, 60% from 2La, 63% from 3La and 67% from 4La.

**Figure 6:**
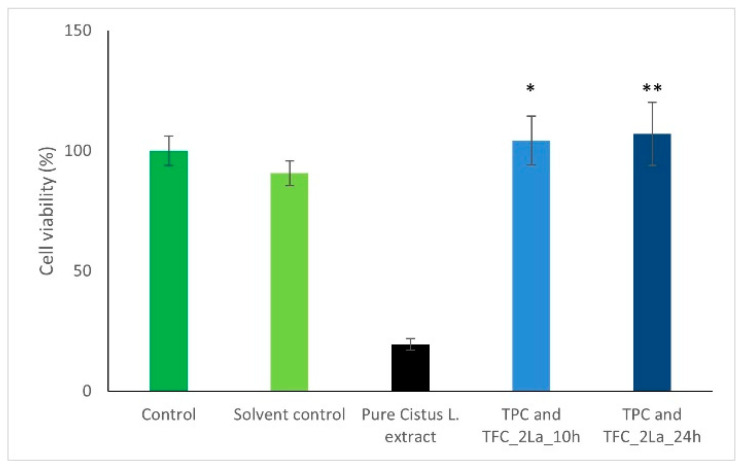
Keratinocytes (Ker-CT) cell line viability measured using 3-(4,5-dimethylthiazol-2-yl)-2,5-diphenyltetrazolium bromide (MTT) assay after 24 h of incubation: Control—keratinocytes cultured in standard medium; Solvent control—control, in which the influence of solvent on viability of keratinocytes was evaluated (medium diluted with water); Pure *Cistus* L. extract—*Cistus* L. extract used for Solution A preparation; total phenolic content (TPC) and total flavonoid content (TFC)_2La_10h—*Cistus* L. extract with the concentration of polyphenols and flavonoids released from plaster after 10 h; TPC and TFC_2La_24h—*Cistus* L. extract with the concentration of polyphenols and flavonoids released from plaster after 24 h. The statistical analysis was performed using one-way ANOVA—Sidak’s test. * *p* ≤ 0.05; ** *p* ≤ 0.01.

It has been positively demonstrated that polyphenols and flavonoids protect human keratinocytes from damages [25,26,27]. However, some investigators have reported that the biologically active compounds extracted from plant materials may also possess carcinogenic or genotoxic effects at high doses or concentrations [28,29]. On the other hand, in a previous paper, we demonstrated that only a slight decrease in BAC concentration was found after steam sterilization performed according to ISO 17665-1: 2006 [9]. Thus, we performed a safety assessment of the TPC and TFC released from 2La plaster with the use of human keratinocytes. Figure 6 shows the MTT assay after 24 h of incubation with extracts obtained from 2La plaster after 10 and 24 h as well as of pure *Cistus* L. extract used for Solvent A preparation and successively exploited for plaster modification.

As is shown in Figure 6, total phenolic and flavonoid content released after 10 and 24 h from 2La plaster has no negative influence on the keratinocytes viability during the incubation period. Moreover, comparing to the Solvent control, the extracts exhibit a significant positive influence on cells viability. They promote proliferation of keratinocytes. 

**Figure 7:**
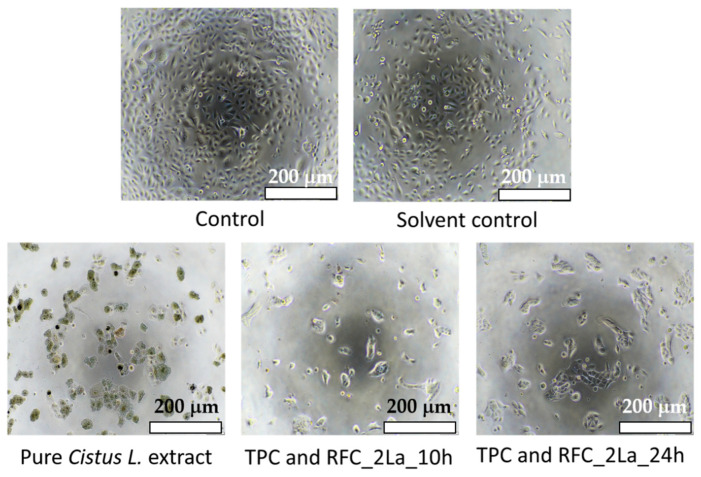
Images of the Ker-CT cell line were obtained with an inverted phase contrast microscope after 24 h of incubation without the *Cistus* L. extract (Control), diluted with water (Solvent control) and with *Cistus* L. extract Pure *Cistus L*. extract, TPC and TFC_2La_10h and TPC and TFC_2La_24h.

In fact, in Figure 7, obtained by means of inverted phase contrast microscope, it could be observed that cells treated with TPC and TFC_2La_10h and TPC and TFC_2La_24h extracts, start to proliferate, extend cellular viability and form colonies typical for epidermal cells. Therefore, it can be expected that designed plasters could improve efficacy in the treatment of skin wounds. Furthermore, we carried out additional investigation on the pure *Cistus L*. extract. The achieved results, both by MTT test and inverted phase contrast microscope observation, confirm that the high concentration of polyphenols and flavonoids has a negative influence on cell viability. On the other hand, this cytotoxic effect can be considered an anti-cancer drug compound and it could be considered in future work.

## 4. Conclusions

In this paper, we investigated the preparation of novel plasters containing BACs, to be used in order to create a moist wound-healing environment, when the skin is damaged because of radiotherapy. A preliminary study showed that the maximum CMC concentration useful for dip-coating was 5 wt.%; moreover, the ratio of CMC/BAC extract which allowed for the preparation of a flexible membrane was found to be 4:1. The plasters were prepared by dip-coating into the BAC/CMC solution, depositing from one to four layers. In the case of one layer, the CMC/BAC extract was unregularly distributed on the plaster fibers, while in plasters with more layers the fibers were covered by a uniform film; the more layers, the thicker the film. On the other hand, plasters containing three and four layers resulted brittle, while the one formed by two layers was still flexible. All the investigated plasters exhibited excellent water wettability. Release experiments showed that a “burst” release occurs in the first hour, while saturation is not yet reached after 24 h; on the other hand, the amount of released polyphenols and flavonoids increases on increasing the number of deposited layers, as expected. Summing up, if we consider the flexibility of the plasters and the amount of released BACs after 24 h, the plaster obtained after two dip-coating steps seems to be an acceptable compromise between comfortable mechanical properties and active principles administration. Preliminary investigations on keratinocytes cultured in standard medium suggest that designed plasters could improve efficacy in the treatment of skin wounds.

## Figures and Tables

**Table 1:** Water static contact angles of neat plaster and samples 2LaW, 2La–4La.

Sample.	CA (°)
Neat plaster	113 ± 9
2LaW	61 ± 4
2La	16 ± 1
3La	19 ± 1
4La	18 ± 2

## Data Availability

The data presented in this study are available on request from the corresponding author. The data are not publicly available due to their deposition at an offline disk, MEMTEC group, URV, Tarragona, Spain.

## Supplementary Materials

The following are available online at https://www.mdpi.com/1996-1944/14/3/582/s1, Figure S1: Calibration curve for determination of DPPH concentration in Radical Scavenging Activity (RSA) assay.
