# Supplementary material for: Medical Plaster Enhancement by Coating with Cistus L. Extracts within a Chitosan Matrix: From Natural Complexity to Health Care Simplicity
Source: Materials (Basel). 2021 Jan 27;14(3):582. doi: 10.3390/ma14030582 (PMC7866121; doi:10.3390/ma14030582)
Supplement: Supplementary file 1 [file materials-14-00582-s001.pdf]

Supplementary information

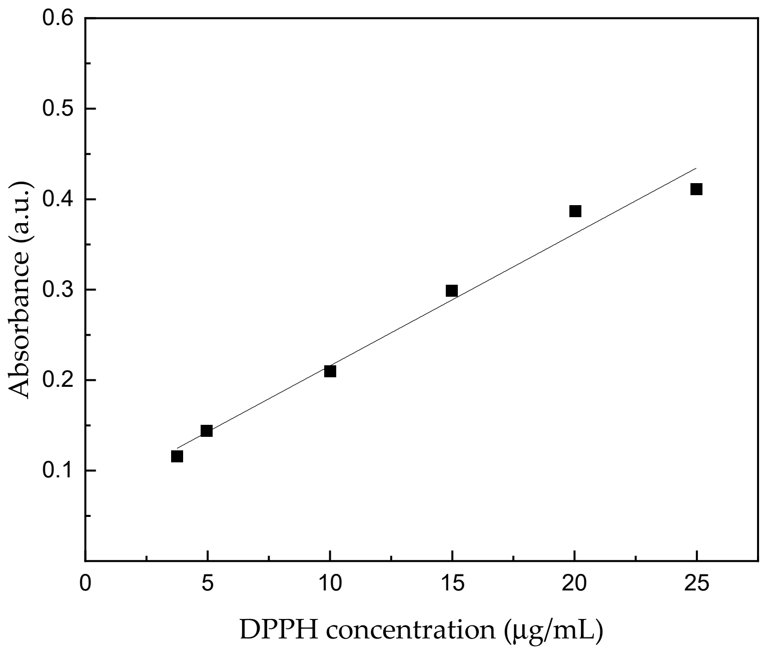

Figure S1. Calibration curve for determination of DPPH concentration in Radical Scavenging Activity (RSA) assay.
